# An oral health optimized diet reduces the load of potential cariogenic and periodontal bacterial species in the supragingival oral plaque: A randomized controlled pilot study

**DOI:** 10.1002/mbo3.1056

**Published:** 2020-05-17

**Authors:** Christian Tennert, Ann‐Christin Reinmuth, Katharina Bremer, Ali Al‐Ahmad, Lamprini Karygianni, Elmar Hellwig, Kirstin Vach, Petra Ratka‐Krüger, Annette Wittmer, Johan Peter Woelber

**Affiliations:** ^1^ Department of Restorative, Preventive and Pediatric Dentistry University of Bern Bern Switzerland; ^2^ Department of Operative Dentistry and Periodontology Faculty of Medicine Center for Dental Medicine Medical Center – University of Freiburg Freiburg im Breisgau Germany; ^3^ Clinic of Preventive Dentistry, Periodontology and Cariology Center of Dental Medicine University of Zürich Zürich Switzerland; ^4^ Department of Medical Biometry and Statistics Medical Center – University of Freiburg Freiburg im Breisgau Germany; ^5^ Department of Microbiology and Hygiene Institute of Medical Microbiology and Hygiene Medical Center – University of Freiburg Freiburg im Breisgau Germany

**Keywords:** fatty acids, omega 3, nutrition, oral microbiota, vitamins, low‐carb diet

## Abstract

This study aimed to investigate the effects of an oral health optimized diet on the composition of the supragingival oral plaque in a randomized controlled trial. Participants of the standard diet group (*n* = 5) had a diet high in processed carbohydrates and did not change their dietary behavior during the observation. The healthy diet group (*n* = 9) had to change the diet after 2 weeks from a diet high in processed carbohydrates to a diet low in carbohydrates, rich in omega‐3 fatty acids, rich in vitamins C and D, antioxidants and fiber for 4 weeks. Saliva and supragingival plaque samples were taken at the end of week two and eight of the observation period to investigate the composition of microbiota in saliva and supragingival plaque. Data were subjected to an exploratory analysis to identify significant differences. Statistically significant differences were only found in the healthy diet group between the baseline (week 2) and the final sample (week 8) for specific species in plaque and saliva samples. A reduction of the total counts of *Streptococcus mitis* group, *Granulicatella adiacens*, *Actinomyces* spp., and *Fusobacterium* spp. was found in plaque samples of the healthy diet group. In saliva samples of the healthy diet group, the total counts of *Actinomyces* spp. and *Capnocytophaga* spp. decreased. A diet low in carbohydrates, rich in omega‐3 fatty acids, rich in vitamins C and D, and rich in fiber reduced *Streptococcus mitis* group*, Granulicatella adiacens, Actinomyces* spp., and *Fusobacterium* spp. in the supragingival plaque.

## Graphical abstract

This study aimed to investigate the effects of an oral health optimized diet on the composition of the supragingival oral plaque in a randomized controlled trial. A diet low in carbohydrates, rich in omega‐3 fatty acids, rich in vitamins C and D, and rich in fiber induces significantly lower counts of caries‐ and periodontitis‐associated pathogens and thus, a favorable shift in the bacterial.

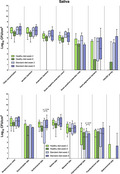

## 1 INTRODUCTION

Caries is the most common disease in the world affecting 2.3 billion people and 560.000 children and can affect humans throughout their lifetime (GBD 2015 Disease and Injury Incidence and Prevalence Collaborators, 2016; Kassebaum et al., 2015). Periodontitis is a complex inflammatory disease resulting from an interplay of bacterial infection and host response to bacterial challenge, and it is modified by local, environmental, and genetic factors (Saini, Marawar, Shete, & Saini, 2009). Caries can be associated with pain. Tooth loss is often caused by severe caries and/or periodontal disease (Edelstein, 2006; Featherstone, 2004). The oral cavity harbors the microbiota of an unknown number of bacteria and fungi (Jenkinson & Lamont, 2005; Paster, Olsen, Aas, & Dewhirst, 2000). To date, about 1,000 different microbial species have been identified (Griffen et al., 2011; Wade, 2013). In 1965, Löe and coworkers found a dental plaque to be an etiological factor for gingival inflammation. They observed increased gingival inflammation when participants discontinued oral hygiene procedures. In a similar experiment, discontinued oral hygiene and added sucrose solution led to rapid caries progression (Von der Fehr, Loe, & Theilade, 1970). The oral microbiome is an extremely diverse, dynamic, and unique ecosystem in the human body (Marsh, 2005). Dental plaque forms in an ordered way involving diverse microbial species remaining relatively stable over time (microbial homeostasis) in healthy individuals (Marsh, 2006). Several environmental factors impact the microbial homeostasis, such as temperature, salinity, oxygen supply, nutrients, pH conditions, and the redox potential, and contribute to the composition of microbial biofilms present at each location (Takahashi & Nyvad, 2011). Changes in environmental factors may lead to changes in the composition of the dental biofilm. Dental caries occurs as a result of a shift in the composition of the oral biofilm. Frequent consumption of fermentable carbohydrates disrupts the microbial homeostasis of the established microbial community by the selection of acidogenic and acid‐tolerant species, which are responsible for caries development (Bowen, 2013). Already in the 1880s, the consumption of fermentable carbohydrates, in particular of sucrose, has been identified to be directly related to the cariogenic potential of microorganisms (Miller, 1884). Caries is mediated by microorganisms belonging to the natural flora of the oral cavity (Cate, 2006; Peterson et al., 2013; Scheie & Petersen, 2004). A high intake of carbohydrates has been shown to promote dysbiosis and chronic inflammatory diseases (Adler et al., 2013; Hujoel, 2009). Reducing the intake of carbohydrates seems to reduce gingival inflammation (Hujoel, 2009). Previous investigations of our group found that an oral health optimized diet significantly reduced gingival and periodontal inflammation in a clinically important range without any changes in oral hygiene performance (Woelber et al., 2016). The aim of this study was to evaluate the effect of the oral health optimized diet, low in fermentable carbohydrates, and rich in omega 3‐fatty acids, vitamins C and D, antioxidants and rich in fiber on the supragingival microflora in a controlled, randomized study. We assumed that this diet would promote homeostasis within the oral biofilm preventing the growth of cariogenic and periodontopathogenic microorganisms by avoiding processed carbohydrates and delivering macro‐ and micronutrients (omega 3‐fatty acids, vitamins C and D, antioxidants) that support the host immune system.

## 2 MATERIALS AND METHODS

### 2.1 Ethics and trial registration

Before patient recruitment, the study protocol was approved by the University of Freiburg Ethics committee (Reference number 338/14) and registered in an international clinical trial register (German Clinical Trials Register; DRKS00006301; https://www.germanctr.de/).

### 2.2 Inclusion criteria

Patients, aged between 18 and 75, with gingivitis (gingival index (GI) > 0.5) and a diet based primarily on carbohydrates were included in this study (Feinman et al., 2015).

### 2.3 Exclusion criteria

Patients fulfilling one or more of the following criteria were excluded from the study: smoking, infectious or severe systemic diseases, intake of antibiotics, antimicrobial agents, for example, chlorhexidine or immunosuppressive drugs 3 months before or during the study period, drugs influencing gingival inflammation or bleeding (e.g., anticoagulants, cortisone), carbohydrate‐ or insulin‐related diseases (e.g., diabetes), pregnancy, or breastfeeding.

### 2.4 Patient recruitment

For this study, 23 patients were screened to find 16 fitting the inclusion criteria. Patients were informed about the study, and gingival inflammation was assessed using the gingival index (GI) by Loe and Silness (1963). Their diet mainly based on carbohydrates was verified by a verbal dietary anamnesis. After receiving written consent, participants were randomly assigned to the healthy diet group (*n* = 10) or the standard diet group (*n* = 6) following a web‐generated randomization list.

### 2.5 Dietary recommendations

**FIGURE 1:**
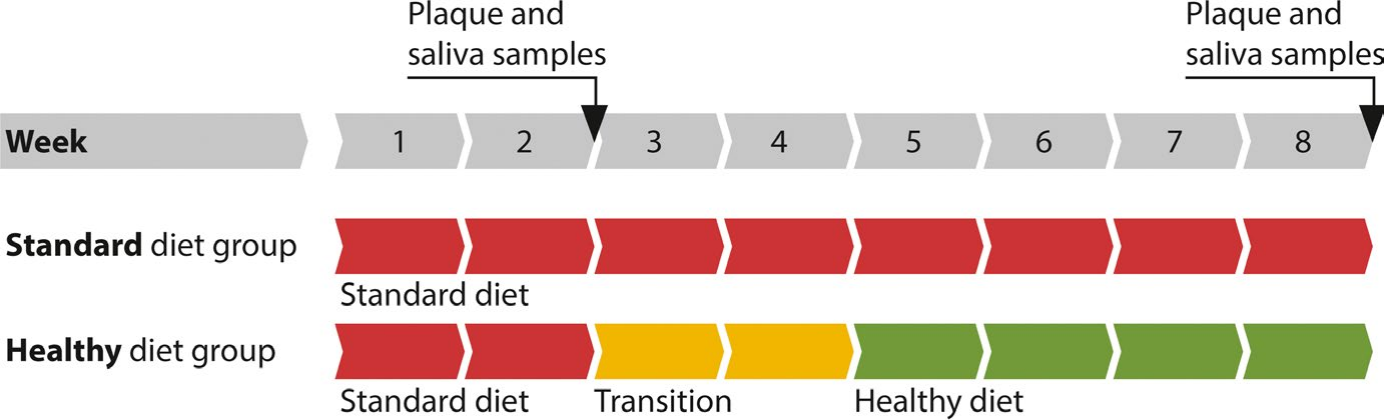
Dietary pattern of both groups. The standard diet group followed a diet primarily based on carbohydrates. The healthy diet group changed their diet after 2 weeks to the health optimized diet for the following 6 weeks, in which the first 2 weeks were a transition period and a strict oral health optimized diet should be performed during the following 4 weeks

Participants were instructed not to change their physical activity and oral hygiene behavior and to stop any interdental cleaning during the entire observation period (8 weeks). All participants had a period of 2 weeks continuing their diet primarily based on carbohydrates and processed foods (“Western diet,” named as standard diet in the study). The participants of the standard diet group (*n* = 6) continued this diet for another 6 weeks. The participants of the healthy diet group (*n* = 10) received nutritional counseling by a nutritional specialist (individual consultation) regarding the oral health optimized diet patterns. The patients were encouraged to follow the oral health optimized diet for the following 6 weeks, in which the first 2 weeks were a transition period and a strict oral health optimized diet should be performed during the following 4 weeks (Figure 1; Woelber et al., 2016).

The oral health optimized diet was based on the current literature concerning diet and general inflammation (Serhan, Chiang, & Dalli, 2015; Woudenbergh et al., 2013), and specifically caries, gingival and periodontal inflammation (Konig, 2000; Serhan et al., 2015; Woudenbergh et al., 2013).

Dietary patterns in the healthy diet group included the following elements as described in our previous study (Woelber et al., 2016):

- Reduction of the intake of carbohydrates to a level <130 g/day, which can be considered as a low‐carb diet (Feinman et al., 2015). This included a restriction in the amount of fructose, disaccharides, sweetened beverages and meals, flour‐containing foods, rice, and potatoes as far as possible. There were no restrictions regarding fruits and vegetables (polysaccharides) as long as the total amount of carbohydrates was considered.
- Daily intake of omega‐3 fatty acids (such as fish oil capsules, a portion of sea fish, and two tablespoons of flaxseed oil), a restriction in the amount of trans‐fatty acids as far as possible (such as fried meals, crisps, donuts, and croissants), and a reduction in omega‐6 fatty acids as far as possible (such as animal products (dairy products, meat, poultry), safflower oil, grape seed oil, sunflower oil, margarine, sesame oil, and corn oil).
- Daily intake of a source of vitamin C (such as two kiwis, one orange, and one bell pepper)
- Daily intake of a source of vitamin D (15 min unprotected in the sun, supplementation with 500 international units [12.5 µg]).
- Daily intake of micronutrients and antioxidants (such as fruits and vegetables, a handful of berries, one cup of green tea, and coffee)
- Daily intake of fiber (fruits and vegetables).

The diet recommendations were given by verbal counseling (30 min) and by handing out an information brochure containing an additional list of restricted and recommended foods and meals.

The participants of both groups had to record their diet using a preset dietary journal. They were advised to write down everything they ate and drank at which time of the day for each day of the observation period. The participant's nutrition diaries were checked at the end of every week to make sure, each participant fulfills the recommended diet in each group, and adjustments could be made, in case the recommendations are not fully achieved. Gingival and periodontal assessment for both groups was performed as described in our previous study (Woelber et al., 2016).

### 2.6 Sampling procedure

All samples were taken by the same examiner. Saliva and supragingival plaque samples were taken at the end of week two and eight of the observation period for both groups (Figure 1).

A saliva sample was taken using a sterile foam pellet by swabbing the lower vestibule. The pellet was transferred into a vial containing 0.75 ml of reduced transport fluid (RTF) (Schirrmeister et al., 2009). The supragingival plaque was collected from all tooth surfaces using a sterile scaler (Anterior scaler, Hu‐Friedy Mfg. Co., LLC.). The collected plaque was then transferred into a vial containing 0.75 ml of RTF. Before and after sampling, the vials were weighed to assess the amount of collected plaque using a laboratory balance (Entris balance, Sartorius Lab Instruments GmbH & Co.KG). The vials were stored at −80°C until microbiological analysis was performed.

### 2.7 Isolation and identification of microorganisms

The supragingival plaque samples were treated for 30 s in an ultrasonic bath on ice. Dilution series (10^–4^–10^–8^) of the plaque and salivary samples were prepared as described earlier in detail (Al‐Ahmad et al., 2010). Each dilution was plated on yeast‐cysteine blood agar plates (HCB) and Columbia blood agar (CBA) plates. The HCB agar plates were used to cultivate anaerobic bacteria at 37°C for 10 days (anaerobic chamber, Anaerocult A, Merck). The CBA plates were incubated at 37°C and 5%–10% CO_2_ atmosphere for 3 days to cultivate aerobic and facultative anaerobic bacteria. The colonies were differentiated by morphology, color, size, and hemolytic reaction and counted to determine the number of colony‐forming units (CFU) per ml. Subsequently, subcultures from all colony types were made to obtain pure cultures from representative colonies. All samples were analyzed considering different dilutions. Bacterial cell morphologies were determined using Gram‐stains and to visualize bacteria using light microscopy (Axioscope, Zeiss; 1,000× magnification). The subsequent identification of the pure bacterial colonies was conducted by MALDI‐TOF MS analysis in a MALDI Biotyper Microflex LT (Bruker Daltonik) according to the manufacturer's instructions and as described earlier in detail (Anderson et al., 2014). The BioTyper 3.0 software was used to compare the obtained spectra with a reference database. The resulting similarity value was expressed as a log score. A score of ≥2.000 was required for identification on the species level, and a score of ≥1.700 indicated identification on the genus level whereas any score under 1.700 resulted in no significant similarity of the obtained spectrum with any database entry. Bacterial isolates that could not be identified by MALDI‐TOF MS unambiguously were characterized by 16S rRNA gene sequencing. Pure bacterial isolates were picked up using a sterile inoculating loop, and the DNA from each isolate was extracted using the QIAamp DNA Mini Kit (Qiagen) according to the manufacturer's instructions. A 16S rRNA gene amplification was performed in a total volume of 50 µl, containing 5 µl 10× PCR‐buffer (Qiagen), MgCl_2_ (2.5 mM), 200 µM of each deoxyribonucleoside triphosphate (dNTP), 2 U Taq Polymerase (Qiagen), and 300 nM of reverse‐ and forward‐primer. For amplification of the 16S rRNA gene, a set of primers (forward‐primer TP16U1: 5′‐AGAGTTTGATC[C/A]TGGCTCAG‐3′ and reverse‐primer RT16U6: 5′‐ATTGTAGCACGTGTGT[A/C]GCCC‐3′) was used as published in a previous study (Schirrmeister et al., 2009). The 1,018 bp‐long PCR products were extracted and purified using the GFX PCR DNA and gel band purification kit (Amersham Biosciences Europe GmbH). Purified PCR products were sequenced by Eurofins Genomics (Ebersberg), and the obtained sequences were analyzed using the BLAST program from the NCBI (http://www.ncbi.nih.gov/BLAST). Sequence comparison to GenBank data entries (>97% similarity) served for the identification of the bacterial strains.

### 2.8 Statistical analysis

The sample size calculation was based on an earlier study (Baumgartner et al., 2009). Accordingly, with a power = 80% and alpha = 5%, *n* = 12 participants would be needed. Due to the assumption of correlated participants (population of family members) in the Baumgartner study (Baumgartner et al., 2009), *n* = 10 participants were aimed for the sample size in this study in an independent population. The control group of *n* = 5 participants was not determined by sample size calculation. The study was considered as a pilot study. Randomization was performed by one of the authors (JPW) using a web‐based randomizer (www.random.org, Randomness and Integrity Services Ltd.) with a presetting of five participants (*n* = 5) for the standard diet group and ten participants (*n* = 10) for the healthy diet group. For a descriptive analysis mean, median and standard deviations (*SD*) were computed. For explorative analysis, the concentrations of bacteria were log‐10‐transformed. Values below the detection limit were set to the detection limit of 4. Paired *t* tests were used to compare values after week 2 and week 8 of the observation period within each group. Because an exploratory data analysis of the results was conducted, a correction for multiple comparisons is not required. An unpaired *t* test for unequal variances was applied to check for differences in changes after week 2 and 8 in the control or experimental group. Tests were only conducted, if more than half of the values in each group were above the limit of detection. The calculations were performed with the statistical software STATA 14.1 (StataCorp).

## 3 RESULTS

Two participants (participant 4 and 16) dropped out. Participant 4 dropped out due to a lack of time for study participation. Participant 16 was not included in the analysis, because the baseline sample was culture‐negative.

The participant's nutrition diaries were checked at the end of every week to make sure, each participant fulfills the recommended diet in each group.

In the healthy diet group (*n* = 9), there were 5 women and 4 men. The mean age in this group was 34.4 years, ranging from 23 to 70 years. In the standard diet group (*n* = 5), there were 3 women and 2 men. The mean age in this group was 34.0 years, ranging from 24 to 63 years (Table 1).

**TABLE 1:** Demographic and clinical data at participant recruitment

Study participant	Age	Sex	Group	PI	GI
1	34	Male	Healthy diet	0.8	1.4
2	29	Female	Healthy diet	0.8	0.8
3	70	Male	Healthy diet	1.9	1.7
4^a^	47	Female	Healthy diet	0.7	0.9
5	32	Male	Healthy diet	1.3	1.1
6	26	Female	Healthy diet	0.5	1.5
7	23	Female	Healthy diet	0.5	0.8
8	27	Female	Healthy diet	1.1	1.6
9	28	Female	Healthy diet	0.5	1.1
10	28	Male	Healthy diet	0.8	1.2
11	32	Male	Standard diet	1.0	1.1
12	63	Female	Standard diet	1.7	1.1
13	24	Female	Standard diet	0.4	0.8
14	25	Male	Standard diet	0.6	1.1
15	26	Female	Standard diet	0.2	0.9
16^a^	36	Male	Standard diet	0.7	1.1

^a^ Dropout.

### 3.1 The healthy diet did not induce any significant changes on the total bacterial counts of dental plaque and saliva

**FIGURE 2:**
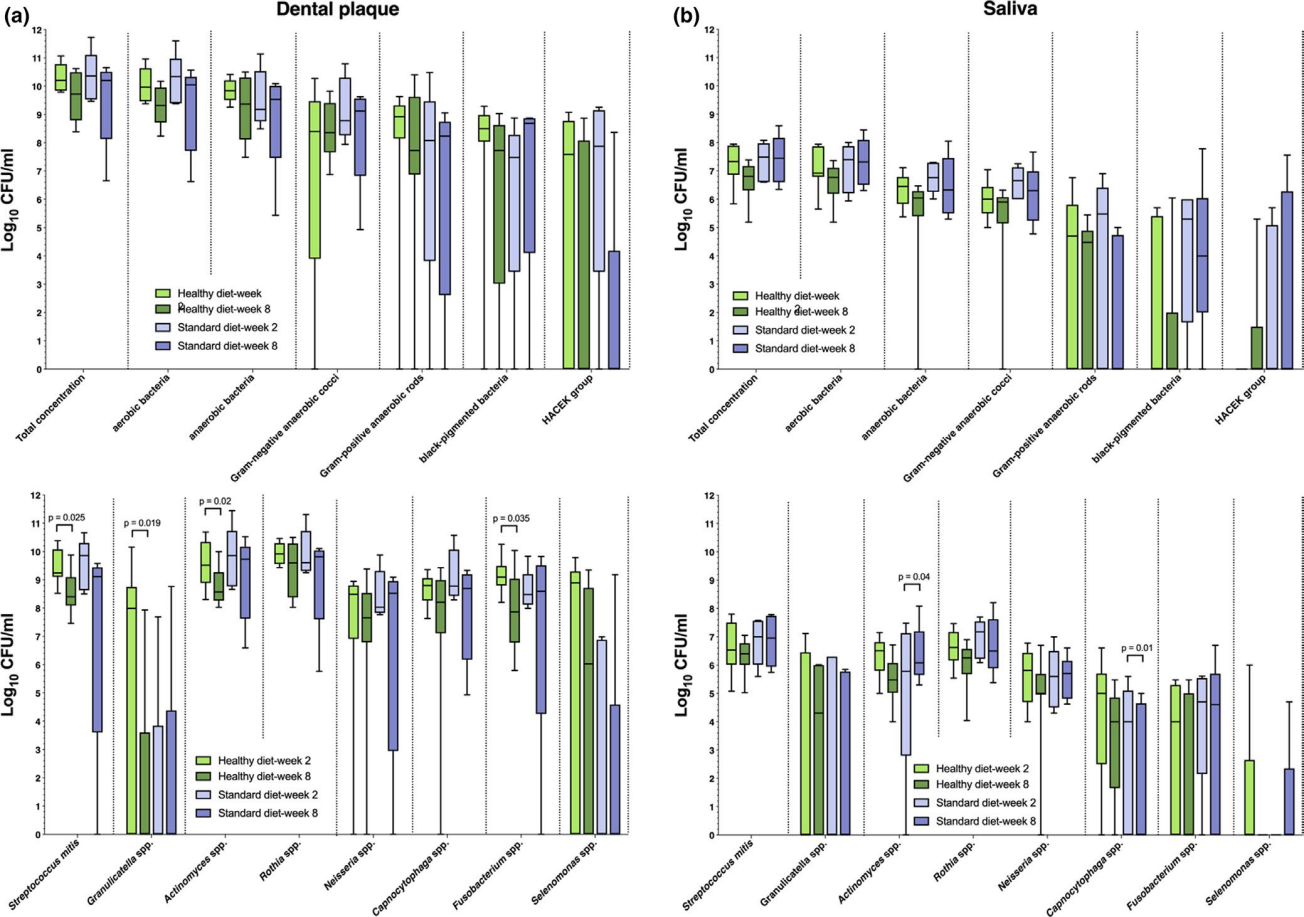
Boxplots of CFU counts, demonstrating the impact of diet on (a) dental plaque and (b) salivary microorganisms among 14 study probands after 2 weeks (baseline) and 8 weeks (final phase) following either a healthy‐ (*n* = 9) or a standard (*n* = 5) diet. Box plots represent the CFUs determined by selective agar plating, while horizontal lines indicate their median values. Undetectable values were ascribed to the lowest detection limit value of the assay to allow for log transformation. The CFUs are presented on a log_10_ scale per square centimeter (log_10_/ml). Error bars indicate from 5% to 95% percentile. The *p*‐values (*t* test) of the significantly different data (*p* ≤ .05) are marked on the graphs

Box plots in Figure 2 demonstrate cell counts as detected in dental plaque samples (2a) and saliva samples (2b) of participants following either a standard or a healthy diet after 2 (baseline) and 8 weeks (final phase), respectively.

In general, a total of 24 different bacterial species could be isolated after both the healthy and standard diet. The median total bacterial count of the plaque samples at baseline (week 2) was 3.4 × 10^10^ CFU/ml in the healthy diet group and 1.2 × 10^11^ CFU/ml in the standard diet group. The median total bacterial count of the final plaque samples at the end of the observation period (week 8) was 1.4 × 10^10^ CFU/ml in the healthy diet group and 1.8 × 10^11^ CFU/ml in the standard diet group. There were no statistical differences between the samples (week 2 versus week 8) or between the groups (standard diet versus healthy diet).

The median total bacterial count of the saliva samples at baseline (week 2) was 3.7 × 10^7^ CFU/ml in the healthy diet group and 4.7 × 10^7^ CFU/ml in the standard diet group. The median overall bacterial count of the saliva samples at the end of the observation period (week 8) was 9.1x10^6^ CFU/ml in the healthy diet group and 9.7 × 10^7^ CFU/ml in the standard diet group.

### 3.2 The healthy diet did not affect the aerobic and anaerobic bacterial counts of dental plaque and saliva

Overall, bacterial counts of aerobic species and anaerobic species showed no statistically significant differences in both groups of dental plaque and saliva samples (Figure 2).

The median aerobic/anaerobic bacterial count of the plaque samples at baseline (week 2) was 10.03/9.84 log_10_ CFU/ml in the healthy diet group and 10.21/9.55 log_10_ CFU/ml in the standard diet group, respectively. The median aerobic/anaerobic bacterial count the final plaque samples at the end of the observation period (week 8) was 9.31/9.18 log_10_ CFU/ml in the healthy diet group and 9.23/8.89 log_10_ CFU/ml in the standard diet group, respectively. There were no statistical differences between the samples (week 2 versus week 8) or between the groups (standard diet versus healthy diet).

The median aerobic/anaerobic bacterial count of the saliva samples at baseline (week 2) was 7.13/6.30 log_10_ CFU/ml in the healthy diet group and 7.11/6.77 log_10_ CFU/ml in the standard diet group, respectively. The median aerobic/anaerobic bacterial count of the saliva samples at the end of the observation period (week 8) was 6.60/5.34 log_10_ CFU/ml in the healthy diet group and 7.30/6.34 log_10_ CFU/ml in the standard diet group.

### 3.3 Reduced counts of *Streptococcus mitis* group, *Granulicatella adiacens*, *Actinomyces* spp., and *Fusobacterium* spp. were detected in dental plaque after a healthy diet

Interestingly, statistically significant differences between the baseline (week 2) and the final sample (week 8) were only found in the healthy diet group for specific species in dental plaque samples (Figure 2a). A significant reduction of *Streptococcus mitis* group (*p* = .025), *Granulicatella adiacens* (*p* = .019), *Actinomyces* spp. (*p* = .02), and *Fusobacterium* spp. (*p* = .035) was found in dental plaque samples of the healthy diet group between the baseline (week 2) and the final phase (week 8).

### 3.4 Counts of *Actinomyces* spp. increased, whereas counts of *Capnocytophaga* spp. decreased in saliva samples after standard diet

In saliva samples, statistically significant differences between the baseline (week 2) and the final sample (week 8) could only be detected in the standard diet group for specific species (Figure 2b). In specific, counts of *Actinomyces* spp. increased significantly (*p* = .04), while the counts of *Capnocytophaga* spp decreased significantly (*p* = .01) between baseline (week 2) and the final phase (week 8).

### 3.5 High inter‐ and intraindividual discrepancies were detected in both standard and healthy diet groups

**FIGURE 3:**
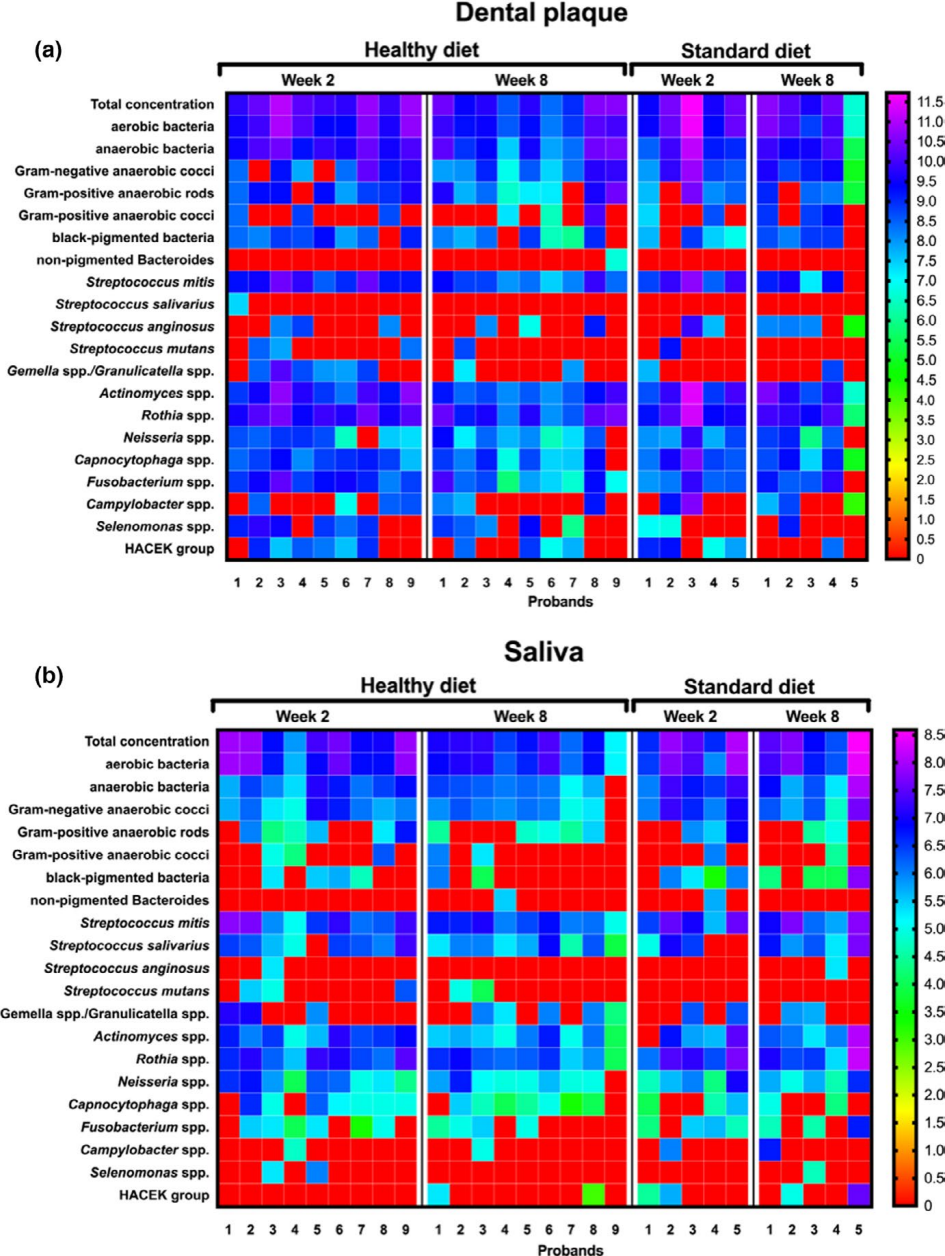
Heatmap demonstrating the absolute distribution (in log_10_/ml) of different bacterial groups/species among 14 study probands after 2 weeks (baseline) and 8 weeks (final phase) following either a healthy‐ (*n* = 9) or a standard (*n* = 5) diet. The influence of diet on the dental plaque‐ and salivary microorganisms after two‐ and eight weeks is demonstrated on panels (a) and (b), respectively. Participant numbers for each treatment group are shown in columns and variables (bacterial groups, species, and genera) in rows. The colors as depicted on the color scale bars on the right vary to indicate data values change for the different samples; Plaque: low: 0–4, moderate: 4–8, high: 8–12; Saliva: low: 0–3, moderate: 3–6, high: 6–9

The Heatmap in Figure 3 shows an overview of the bacterial counts for each participant as detected in dental plaque samples (a) and saliva samples (b) upon either a standard or a healthy diet after two (baseline) and eight weeks (final phase), respectively.

## 4 DISCUSSION

In the present pilot study, the conditions of an oral health optimized diet seem to significantly reduce several species in dental plaque and saliva. Significant reductions of *Streptococcus mitis* group species, *Granulicatella adiacens*, *Actinomyces* spp., and *Fusobacterium* spp. were found in plaque samples, and *Actinomyces* spp. and *Capnocytophaga* spp. were significantly reduced in saliva samples of the healthy diet group after 4 weeks of performing an oral health optimized diet. *Streptococci* from the mitis group and *Actinomyces* spp. are species producing acids. *Streptococcus mitis group* ferment glucose, maltose, sucrose, lactose, and salicin to produce acids and some strains can also ferment raffinose and trehalose producing acids (Gross et al., 2012). The reduction of carbohydrates in general and especially in processed carbohydrates in the healthy diet group may lead to a nutrient deficiency for these species. This may reduce the overall risk of caries for the patient. *Actinomyces* strains are predominant in periodontal tissues in both healthy and inflammatory conditions (Socransky & Haffajee, 2000; Vielkind, Jentsch, & Eschrich, 2015). *Granulicatella adiacens* species are catalase‐negative, Gram‐positive cocci, and these bacteria are normal commensals of human mucosal surfaces, inhabiting the oral cavity, urogenital and gastrointestinal tract and only rarely causing diseases (Mikkelsen, Theilade, & Poulsen, 2000; Sato, Kanamoto, & Inoue, 1999). It is a potential virulent species as *G. adiacens* is a cause for endocarditis and a spectrum of other infections (Gardenier, Hranjec, Sawyer, & Bonatti, 2011).

In our previous study, we found that an oral health optimized diet significantly reduced gingival and periodontal inflammation in a clinically important range (Woelber et al., 2016). The analysis of clinical results of the microbial composition in this study revealed some changes in potentially pathogenic bacteria causing gingivitis and periodontitis. However, the change in diet in the healthy diet group might also lead to changes in complex metabolic pathways resulting in lower metabolic activity of pathogenic bacteria. Furthermore, the higher intake in anti‐inflammatory components, such as omega‐3 fatty acids, vitamins (especially vitamins C and D), minerals and antioxidants, and the reduction of pro‐inflammatory nutritional components (omega‐6 fatty acids, reduction of carbohydrate intake, especially sugars, simple carbohydrates, and flour products) was suggested to enhance the hosts immune system, reducing inflammation and thus, reducing the amount of nutrients for pathogenic bacteria (Aizawa et al., 2009; Lingstrom, van Houte, & Kashket, 2000; Woudenbergh et al., 2013).

In this study, the culture technique was used. This might have underestimated the diversity of the microbiota in the oral biofilm samples as it is known that culture‐dependent methods, in general, detect only up to 50% of the oral microbiota (Wade, 2004, 2013). Furthermore, the proportion of identified species can be far <50% of the total viable number which can be determined on different culture media. Nevertheless, the culture technique reveals only viable and active bacteria as compared to a microbiome analysis which is based on DNA sequencing. Additionally, isolated bacteria can be a target for further studies such as virulence analysis and biofilm formation capacity. Though OMICS research has been greatly informative, problems related to study design, data quality, integration, and reproducibility still need to be addressed (Nascimento, Zaura, Mira, Takahashi, & Ten Cate, 2017). Furthermore, the authors emphasized the need for continuous updates of the modern computationally demanding and culture‐independent technologies which require expertise in advanced bioinformatics for reliable interpretation of data. Hence, a combination of microbiological analysis methods including culture technique and high throughput sequencing should be used in future studies to confirm and complement the data presented in this study.

## 5 CONCLUSIONS

A diet rich in anti‐inflammatory components and low in carbohydrates especially processed carbohydrates led to changes in the composition of oral microbiota compared to a “western diet.” A diet low in carbohydrates, rich in omega‐3 fatty acids, rich in vitamins C and D, and rich in fiber reduced *Streptococcus mitis* group*, Granulicatella adiacens, Actinomyces* spp. and *Fusobacterium* spp. in the supragingival plaque.

## 6 ETHICS STATEMENT

All procedures performed in studies involving human participants were following the ethical standards of the institutional and national research committee and with the 1964 Helsinki declaration and its later amendments or comparable ethical standards. Before participant recruitment, the study was approved by the University of Freiburg Ethics committee (Reference number 338/14) and registered in an international clinical trial register (German Clinical Trials Register; https://www.drks.de/drks_web/navigate.do?navigationId=trial.HTML&TRIAL_ID=DRKS00006301). All participants gave their written consent.

## CONFLICTS OF INTEREST

None declared.

## AUTHOR CONTRIBUTION

**Christian Tennert:** Conceptualization (lead); Formal analysis (equal); Methodology (equal); Project administration (equal); Supervision (equal); Writing‐original draft (equal); Writing‐review & editing (equal). **Ann‐Christin Reinmuth:** Data curation (equal); Formal analysis (equal); Investigation (equal); Visualization (equal). **Katharina Bremer:** Data curation (equal); Investigation (equal). **Ali Al‐Ahmad:** Conceptualization (equal); Funding acquisition (equal); Methodology (equal); Writing‐review & editing (equal). **Lamprini Karygianni:** Formal analysis (equal); Visualization (equal); Writing‐review & editing (equal). **Elmar Hellwig:** Resources (lead); Supervision (equal); Writing‐review & editing (equal). **Kristin Vach:** Formal analysis (lead); Methodology (equal); Writing‐review & editing (equal). **Petra Ratka‐Krüger:** Project administration (equal); Supervision (equal); Writing‐original draft (equal). **Annette Wittmer:** Data curation (equal); Investigation (lead); Writing‐review & editing (equal). **Johan Peter Woelber:** Conceptualization (equal); Methodology (equal); Project administration (equal); Supervision (equal); Writing‐review & editing (equal).

## Data Availability

The datasets used and analyzed during the current study are available from the corresponding author on reasonable request.
